# A Computationally Efficient Online/Offline Signature Scheme for Underwater Wireless Sensor Networks

**DOI:** 10.3390/s22145150

**Published:** 2022-07-08

**Authors:** Syed Sajid Ullah, Saddam Hussain, Mueen Uddin, Roobaea Alroobaea, Jawaid Iqbal, Abdullah M. Baqasah, Maha Abdelhaq, Raed Alsaqour

**Affiliations:** 1 Department of Information and Communication Technology, University of Agder (UiA), N-4898 Grimstad, Norway; syed.s.ullah@uia.no; 2 Department of Electrical and Computer Engineering, Villanova University, Villanova, PA 19085, USA; 3 School of Digital Science, Universiti Brunei Darussalam, Jalan Tungku Link, Gadong BE1410, Brunei; 4 College of Computing and IT, University of Doha for Science and Technology, Doha 24449, Qatar; mueenmalik9516@gmail.com; 5 Department of Computer Science, College of Computers and Information Technology, Taif University, P.O. Box 11099, Taif 21944, Saudi Arabia; r.robai@tu.edu.sa; 6 Department of Computer Science, Capital University of Science and Technology, Islamabad 44000, Pakistan; jawaid5825@gmail.com; 7 Department of Information Technology, College of Computers and Information Technology, Taif University, P.O. Box 11099, Taif 21944, Saudi Arabia; a.baqasah@tu.edu.sa; 8 Department of Information Technology, College of Computer and Information Sciences, Princess Nourah bint Abdulrahman University, P.O. Box 84428, Riyadh 11671, Saudi Arabia; msabdelhaq@pnu.edu.sa; 9 Department of Information Technology, College of Computing and Informatics, Saudi Electronic University, Riyadh 93499, Saudi Arabia; r.alsaqor@seu.edu.sa

**Keywords:** underwater wireless sensor networks, certificateless online/offline signature, authentication scheme

## Abstract

Underwater wireless sensor networks (UWSNs) have emerged as the most widely used wireless network infrastructure in many applications. Sensing nodes are frequently deployed in hostile aquatic environments in order to collect data on resources that are severely limited in terms of transmission time and bandwidth. Since underwater information is very sensitive and unique, the authentication of users is very important to access the data and information. UWSNs have unique communication and computation needs that are not met by the existing digital signature techniques. As a result, a lightweight signature scheme is required to meet the communication and computation requirements. In this research, we present a Certificateless Online/Offline Signature (COOS) mechanism for UWSNs. The proposed scheme is based on the concept of a hyperelliptic curves cryptosystem, which offers the same degree of security as RSA, bilinear pairing, and elliptic curve cryptosystems (ECC) but with a smaller key size. In addition, the proposed scheme was proven secure in the random oracle model under the hyperelliptic curve discrete logarithm problem. A security analysis was also carried out, as well as comparisons with appropriate current online/offline signature schemes. The comparison demonstrated that the proposed scheme is superior to the existing schemes in terms of both security and efficiency. Additionally, we also employed the fuzzy-based Evaluation-based Distance from Average Solutions (EDAS) technique to demonstrate the effectiveness of the proposed scheme.

## 1. Introduction

Currently, there has been a growing interest in monitoring marine ecosystems for scientific research, military applications, and commercial exploitation [1]. The UWSN is the most effective method of monitoring the marine environment. In principle, the UWSN is a wireless communication network comprised of tens or hundreds of battery-powered sensor nodes [2]. Unlike wireless connections between ground sensors, the underwater channel has a high latency and low bandwidth, which uses a lot of power. In addition, changing or recharging a battery in UWSNs is far more complex than in ground WSNs. That is why the current security algorithms struggle with power usage [3]. Due to the constrained resources, the sensor nodes suffer from an energy consumption problem [4]. Therefore, almost all of the existing research and technology on UWSNs is focused on power savings at the expense of security and capability.

Security is one of the key elements in the design of the UWSNs’ protocol and mechanism. As a result of their low cost and proximity to the events they monitor, sensor nodes are prime targets for malicious attacks of many kinds. In addition, the public communication channel makes it possible for any device to participate in the flow of information. Therefore, an attacker might easily control the sensors and unsecured UWSN communication lines. The research available on UWSNs focuses on self-organization, communication, flexibility, low power consumption, and adaptability. Unfortunately, the current studies have a lot of limitations when it comes to how well UWSNs can resist security threats, because resources are very limited, and the security situation is usually server-based because of certain data and communication sites [5].

In the context of security, authentication is necessary. Global WSN authentication solutions, such as public-based RSA [6] and Blom’s symmetric matrix multiplication algorithm [7], have been presented, but they do not work for UWSNs because of their increased computational and communicational complexity. As a result, UWSNs require the development of an authentication system based on signatures [8].

A digital signature is a common solution for ensuring data authenticity in UWSNs. However, traditional digital signature schemes are based on expensive scaler point multiplication of the ECC, hyperelliptic curve devisor multiplication, and bilinear pairing operations, limiting their transmission to resource-limited devices such as sensors and IoT devices. An alternate solution to the problem is to utilize an offline/online signature, where the signature process is divided into online and offline phases. The offline phase performs computationally intensive tasks, while the online phase produces the signature on the message in real time. When installed on UWSNs, the gateway can simplify the online signature to generate authentic messages. Reducing the communication bandwidth and computation time is the key to the actual use of an online/offline signature technique. However, ensuring both the security and effectiveness of an online/offline approach in the real world remains a challenge. This is the main focus of the current paper.

### 1.1. Motivation and Contributions

The computation time and communication overhead are inversely related to the hardness of the underlying security concerns that must be spent on signature formation. Traditional signature techniques such as RSA and bilinear pairing, both of which are based on sub-exponential issues, need a significant amount of computation time and communication overhead and are not suitable for devices that have limited resources. Elliptic curve cryptography (ECC) is utilized instead. Their fundamental issue is a fully exponential one, and it is possible to generate their signatures in a significantly shorter amount of time.

However, it is still challenging to find a cryptographic solution that is appropriate for UWSNs. There are hardly any articles that concentrate on the cryptographic security and privacy for UWSNs [9,10,11,12,13,14]. However, bilinear pairing with elliptic curves is used to apply authenticity in various environments [15]. Since HEC has a higher efficiency and a shorter key length than ECC, bilinear pairing, and RSA, it is often regarded as the most compact and effective form of cryptographic mechanisms. In the proposed work, we focused on proposing a new security solution for UWSNs devices by dividing our algorithm into online and offline phases to further reduce the computational time and communication bandwidth during the device operation. The contributions to this paper are as follows:

- Firstly, we propose a new certificateless online/offline signature scheme based on a hyperelliptic curve cryptosystem for underwater wireless sensor networks.
- Secondly, we present the generic syntax of the proposed certificateless online/offline signature scheme for underwater wireless sensor networks.
- Thirdly, we provide the mathematical construction for the proposed certificateless online/offline signature scheme for underwater wireless sensor networks. The construction is actually an extension of the syntax. The designed approach offers the security necessity of unforgeability against both type one and type two adversaries, an antireplay attack.
- Finally, we compared the computational and communicational overhead of our proposed method with earlier certificateless online and offline signature solutions. According to the findings, the proposed strategy uses significantly fewer computing and communication resources than earlier solutions.

### 1.2. Paper Organization

In the upcoming section (i.e., Section 2), we will review the existing literature. Section 3 presents our proposed network and the construction of an online/offline signature for UWSNs. Section 4, presents the deployment of the proposed scheme on UWSNs. Section 5 presents the formal security analysis and Section 6 added the performance analysis. Section 7 is a review of our contributions while Section 8 concludes the research.

## 2. Related Works

Related studies have been presented to secure the UWSNs in recent years [9,10,11,12,13,14]. Unfortunately, the present key management and cryptographic solutions have some common problems, including computational and communicational complexity and the expansion of ciphertext [4]. Therefore, in the proposed approach, we considered an online/offline signature with a lightweight hyperelliptic curve cryptosystem to reduce the computational and communicational complexities for UWSN communications. Table 1 summarizes the related works.

Evan, Goldreich, and Micali [16] proposed the online/offline signature concept in 1990. The authors divided the signing algorithm into two phases: online and offline. In the absence of a message, heavier computations are transferred to the offline phase, while lighter computations are performed online. During the production process or whenever the device’s power is connected, offline action can be conducted on the background computation device. Shamir and Thuman [17] refined the Trapdoor hash function-based online/offline signature technique in 2001. This improves the online efficiency. However, the technique increases the signature costs and has a trapdoor leak issue. In 2007, Chen [18] created an online/offline signature system employing the dual trapdoor hash function. However, in normal situations, neither method works.

Recently, Liu et al. [19] proposed an identity-based online/offline signature using the elliptic curve discrete logarithm problem (ECDLP). Addobea et al. [20] proposed COOS for mobile health devices in 2020. This study aims to reduce the computational and communication resources required by mobile health devices. According to Xu and Zeng [21], the propose scheme of Addobea et al. [20] is unable to accomplish correctness, a key security property that should be provided by a signature scheme. In the same year, Khan et al. [22] provided a new COOS solution for IoHT employing hyperelliptic curve discrete logarithm problem hardness (HCDLP). According to Hussain et al. [23], the given approach of Khan et al. [22] is insecure when subject to adaptive chosen message attacks. It has been proven that an adversary can fake a valid signature on a message by substituting their own public key in place of the one that is supposed to be used. An attribute-based online/offline signature system for mobile crowdsourcing was presented in 2021 by Hong et al. [24]. Sadly, the authors did not present a mathematical or network model. The solution is theoretical.

**Table 1:** Summary of the literature.

Authors Name & Reference No.	Advantages	Limitations
Liu et al. [19]	Propose an identity-based online/offline signature. The authors utilized ECC to minimize the cost consumptions.	Suffers from key escrow problem The cost consumptions can be reduced further
Addobea et al. [20]	Propose COOS for mobile health devices in 2020. Aims to reduce the computational and communication resources required by mobile health devices.	Suffers from high computational and communicational resource due to heavy bilinear pairing operations. Unable to accomplish correctness [21]
Khan et al. [22]	Propose a new COOS solution for IoHT. Reduced the computational and communicational resources utilizing HCDLP.	Insecure when subject to adaptive chosen message attacks [23]
Hong et al. [24]	Present an online/offline signature system for mobile crowdsourcing.	The authors did not present a mathematical or network model.

The above schemes are based on sophisticated cryptographic methods, i.e., bilinear pairing and ECC, and thus combined with the high cost of computation and communication. These approaches are therefore not compatible with UWSNs equipped with minimal computation and communication resources. To construct an effective cryptographic solution for UWSNs that requires minimal computational resources, there is a critical need for a more concrete and efficient online/offline signature scheme. Our design scheme is based on the HCC, which is a generalized form of an elliptic curve.

## 3. Construction of the Proposed Scheme

### 3.1. Security Threats

In certificateless public key cryptography, two types of adversaries are considered i.e., type-1 ($T_{1}$) and type-2 ($T_{2}$).

The certificateless signature scheme has a unique security concept in comparison to those used by traditional signature schemes. According to the definitions found in [25], a certificateless signature scheme ought to take into account two distinct kinds of adversaries: a Type-I ($T_{1}$) adversary and a Type-II ($T_{2}$) adversary. The adversary $T_{1}$ is meant to stand in for a typical threat posed by a third party against the certificateless signature scheme. This means that $T_{1}$ does not have access to the master key, but it is able to request public keys and replace existing public keys with values of its choosing. The adversarial $T_{2}$ is a representation of a malicious Key Generation Center (KGC) that is responsible for generating users’ partial private keys. It is permissible for the adversary $T_{2}$ to have access to the master key, but they are not authorized to replace the target user’s public key.

### 3.2. Hyperelliptic Curve Cryptosystem (HEC)

Koblitz [26] is the one who first introduced the hyperelliptic curve cryptosystem (HEC), which belongs to a class of algebraic curves. It is also possible to think of it as a more generalized version of the elliptic curves cryptosystem (ECC) [27]. The HEC points, as opposed to ECC points, cannot be obtained from a group in any way [28]. The additive Abelian group that can be generated from a devisor is the subject of computation by the HEC. In comparison to RSA, bilinear pairing, and ECC, the HEC’s parameter size is significantly smaller while maintaining the same level of security. This makes the HEC appealing to resource-constrained devices.

**Figure 1:**
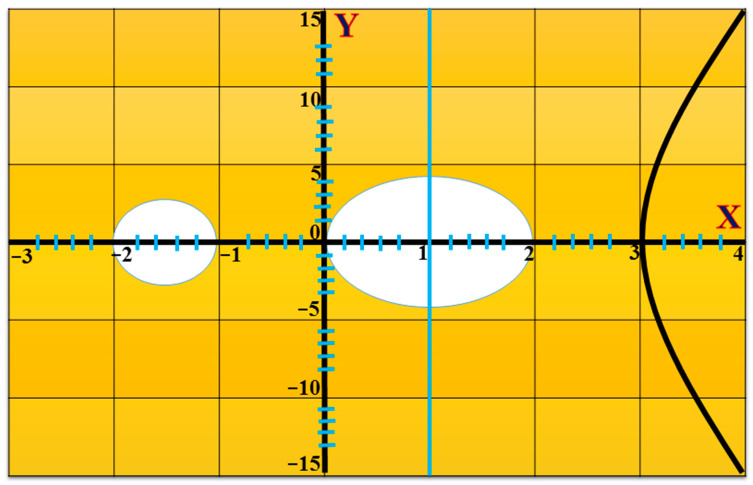
Hyperelliptic curve (genus = 2).

The curve whose genus value is 1 is typically referred to as the ECC curve. Figure 1 [29] illustrates a HEC that has a genus that is higher than 1. In a similar manner, the group order of the finite field ($F_{𝕢}$.) for the (genus = 1) needed operands that were 160 bits long, which necessitated the need for at least $𝕘\;.{𝕝𝕠𝕘}_{𝟚}\left(𝕢\right)\;\approx \;{𝟚}^{𝟙𝟞𝟘}$, where g is the genus of the curve over, $F_{𝕢}$., which is the set of a finite field of order q. In a similar manner, the curve with a genus equal to two needed operands that were 80 bits long. In addition, the curves with a genus equal to three required operands were 54 bits in length [30].

Let us assume that F is a finite field and that $\bar{F}$ is the algebraic closure of F. An HEC of a genus (𝕘 > 1) over F is a set of solutions to the following equation of the curve in the form ( 𝕩,𝕪) ℇ $\bar{F}$ *x* $\bar{F}$.

$$\mathrm{HEC}:\;y^{2}+h\left(x\right)y=f\left(x\right)$$

If there are no pairs of (𝕩, 𝕪) ℇ $\bar{F}$ *x* $\bar{F}$ that satisfy the condition, then the curve in question is regarded to be nonsingular. In addition, the curve in question must be able to satisfy both the previously mentioned curve equation, as well as the subsequent given partial differential equation.

$$2y+h\left(x\right)=0\;\mathrm{and}\;h^{\prime }\left(x\right)y-f^{\prime }\left(x\right)=0$$

The polynomial h(x) ℇ F[u] is a degree of 𝕘, and h(x) ℇ F[u] is the monic polynomial of degree 2𝕘+1.

### 3.3. Complexity Assumptions

During the course of the investigation, we found it necessary to presume the following assumptions:

$F_{𝕢}$ is a finite field with order 𝕢, where $\;𝕢\approx \;{𝟚}^{𝟠𝟘};$
D is a divisor of a HEC, which is a finite sum of points;
D = ∑ $p_{i}ℇ$ HEC $m_{i}$ $p_{i}$, where $m_{i}\;ℇ$ $F_{𝕢}$.

#### 3.3.1. Definition 1. Hyperelliptic Curve Discrete Logarithm Problem (HCDLP)

We made the following supposition for HECDLP.

Let η∈{𝟙,𝟚,𝟛,…,(𝕟−𝟙)} and W=η.D; then, finding η from W is called HCDLP.

#### 3.3.2. Definition 2. Hyperelliptic Curve Computational Diffie-Hellman Problem (HCCDH)

For HCCDHP, we make the following suppositions.

Let η,Υ ∈{𝟙,𝟚,𝟛,…,(𝕟−𝟙)} and W=η.D, T=Υ.η.D; then, finding η from W and Υ from T is called HCCDH.

### 3.4. Network Model

**Figure 2:**
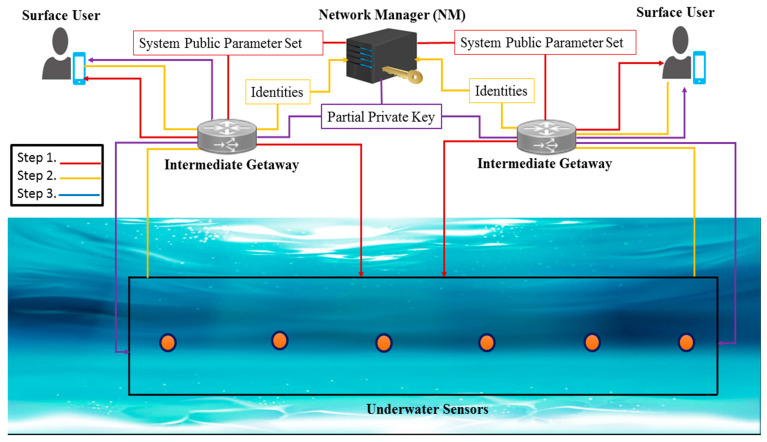
Proposed network model.

In Figure 2, we present the proposed network model for the online/offline sig-nature scheme for the underwater wireless sensors network. The proposed network model consists of a Network Manager (NM), an Intermediate Getaway, Underwater Sensors, and Surface Users.

- Network Manager (NM): It is the responsibility of the NM to establish a secure connection between all of the entities within the networks, and it is a third party that can be trusted.
- Underwater Sensors: These are the sensors that sense the underwater environment and transmit data to the surface of the water.
- A surface user is a device or a client that is interested in underwater sensors, such as an Internet of Things device or a client.
- Intermediary Getaway: The intermediate getaway is a collection of nodes that act as a conduit for data and requests between different entities.

The NM is in charge of the registration process that takes place prior to the creation of communication links. The NM first registers the communication parties in order to facilitate secure communication. A great amount of processing power, memory, and computational capability are available on the intermediate gateway device. Sensors with limited resources collect data and pass it to the intermediary gateway, which then processes it. In the presence of a message, the intermediate gateway then goes through the process of signature generation on the message.

### 3.5. Proposed Online/Offline Signature Algorithm for UWSNs

**Figure 3:**
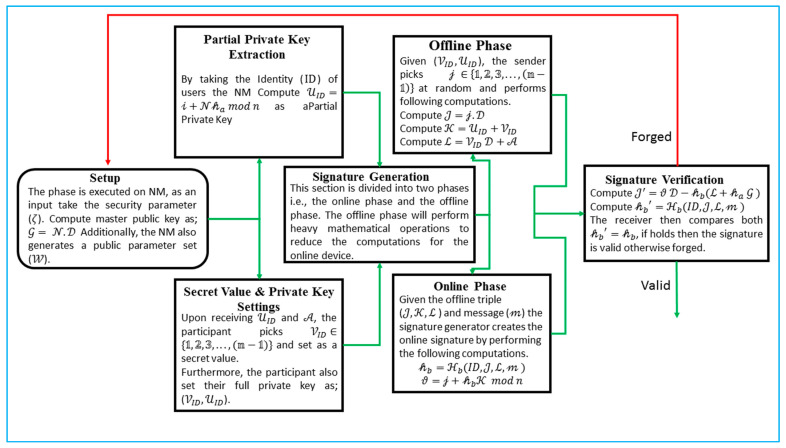
Flowchart of the proposed algorithm.

The symbols that were used in the construction of the proposed online/offline signature algorithm are listed in Table 2 of the following section. Additionally, Figure 3 presents the flowchart of the proposed algorithm.

**Setup:** The phase is carried out on NM, it take the security parameter (ζ) as an input. In addition, the NM will carry out the following procedures in order to produce a public parameter set designated as “(W)”.

- Select the genus (g=2) of HCC with the key size of 80 bits;
- Select N ∈{𝟙,𝟚,𝟛,…,(𝕟−𝟙)} to compute the master public key as G= N.D, where D is a devisor of the hyperelliptic curve cryptosystem (HCC);
- Choose two one-way hash functions ${ℋ}_{a},{ℋ}_{b}$;
- Finally, the NM advertise $W=\{\mathrm{HCC},{ℋ}_{a},{ℋ}_{b},G,n$, D } in the entire network while keeping the N with itself.

**Partial Private Key Extraction:** By taking the identity (ID) of users, the NM perform the following computations:

- First pick 𝒾∈{𝟙,𝟚,𝟛,…,(𝕟−𝟙)};
- Compute A=𝒾.D;
- $𝒻={ℋ}_{a}\left(ID,A\right)$;
- Compute $U_{ID}=𝒾+N𝒻\;mod\;n$.

The NM then send $U_{ID}$ and 𝓐 to the participants. Upon receiving them, the participants can check the validity of the equation as

$$U_{ID}.𝓓=𝓐+G{𝒽}_{a}$$

The partial private key is legitimate if the aforementioned equation is true; else, it is invalid.

**Secret Value and Private Key Settings:** Upon receiving $U_{ID}$ and A, the participants pick $V_{ID}\in \left\{𝟙,𝟚,𝟛,\ldots ,\left(𝕟-𝟙\right)\right\}$ and set it as a secret value.

Furthermore, the participants also set their full private key as ($V_{ID},U_{ID}$).

**Signature Generation:** This section is divided into two phases, i.e., the online phase and the offline phase. The offline phase will perform heavy mathematical operations to reduce the computation for the online phase.

Offline Phase: Given ($V_{ID},U_{ID}$), the sender picks 𝒿 ∈{𝟙,𝟚,𝟛,…,(𝕟−𝟙)}  at random and performs the following computations.

- Compute J=𝒿.D;
- Compute $K=U_{ID}+V_{ID}$;
- Compute $ℒ=V_{ID}\;D+A$.

The triple (J,K,ℒ ) is then assigned to the online phase.

Online Phase: Given the offline triple (J,K,ℒ), fresh nonce (𝜏) and message (𝓂), the signature generator creates an online signature by performing the following computations.

$${𝒽}_{b}={ℋ}_{b}\left(ID,J,ℒ,\tau ,\;𝓂\right)$$

$$\vartheta =𝒿+{𝒽}_{b}K\;mod\;n$$

Finally, the sender computes the triple of ($ℒ,{𝒽}_{b},\vartheta$) as a full signature.

**Signature Verification:** For an identity (ID) and message (𝓂) with the computed signature triple ($ℒ,{𝒽}_{b},\vartheta$) on 𝓂, the receiver verifies the signature by performing the following operations:

- Compute $𝒻={ℋ}_{a}\left(ID,A\right);$
- Compute $J^{\prime }=\vartheta \;D-{𝒽}_{b}\left(ℒ+{𝒽}_{a}\;G\;\right)$;
- Compute ${𝒽}_{b}{}^{\prime }={ℋ}_{b}\left(ID,J^{\prime },ℒ,\tau ,𝓂\;\right)$.

The receiver then compares both ${𝒽}_{b}{}^{\prime }={𝒽}_{b}$; if it holds, then the signature is valid; otherwise, it is forged.

The consistency can be proved from the following equation.

$$=>\;J^{\prime }=\vartheta \;D-{𝒽}_{b}\left(ℒ+{𝒽}_{a}\;G\right)$$

## 4. Deployment of the Proposed Scheme

**Figure 4:**
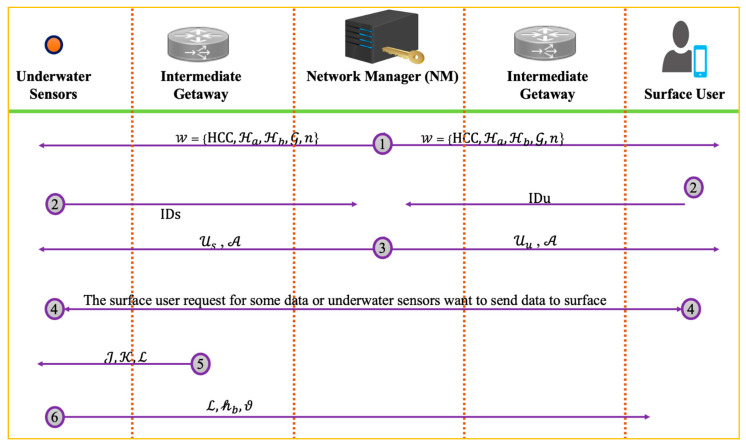
Deployment of the proposed scheme.

For deployment, we consider underwater sensors, and surface users want communication to share data. In this communication, there will be other entities like NM and the intermediate getaway. To make a connection and authentic sources of data, each entity will follow the following steps of the suggested online/offline signature. Figure 4 shows the deployment of the proposed scheme.

### 4.1. Setup, Connectivity, and Keys Extraction

To connect devices, the NM as an input takes the security parameter (ζ), and the KGC generates a public parameter set (W). For this, the NM select a genus (g=2) of HCC with a key size of 80 bits, select N ℇ {1,2,3,4,5,…,(n−1)}, compute the master public key as G= N.D, where D is a devisor of the hyperelliptic curve cryptosystem (HCC), and choose two one-way hash functions ${ℋ}_{0},{ℋ}_{1}.\;$ Finally, the NM advertise $W=\{\mathrm{HCC},{ℋ}_{a},{ℋ}_{b},G,n$} in the entire network while keeping the N with itself.

To contact the network, the underwater sensors and surface user send their identities (IDs, IDu) to NM. By taking the IDs, IDu, the NM first pick 𝒾 ℇ {1,2,3,4,5,…,(n−1)}, compute A=𝒾.D, ${𝒽}_{a}={ℋ}_{a}\left(ID,A\right),$ and compute $U_{i}=𝒾+N{𝒽}_{a}\;mod\;n.$ The NM then send $U_{i}$ and A to the underwater sensors and surface user as a partial private key. Upon receiving it, the users can check the validity $U_{i}\;$ of the equation as $U_{i}.𝓓=𝓐+G{𝒽}_{a}$. If this equation holds, then the partial private key is valid; otherwise, it is invalid. Upon receiving $U_{i}$ and A, the participant picks $V_{i}ℇ\;\left\{1,2,3,4,5,\ldots ,\left(n-1\right)\right\}$ and set it as a secret value. Furthermore, the underwater sensors and surface user also set their full private key as ($V_{i},U_{i}$).

### 4.2. Signature Generation

In this step, the underwater sensors generate the signature on data. As we know, the underwater sensors have limited energy. This section is divided into two phases, i.e., the online phase and the offline phase of the signature. The offline phase will perform heavy mathematical operations to reduce the computations for the online device. The heir of the intermediate gateway performs the offline phase and underwater sensors online phase. The intermediate gateway picks 𝒿 ℇ {1,2,3,4,5,…,(n−1)} at random, computes J=𝒿.D, computes $K=U_{i}+V_{i},$ and computes $ℒ=V_{i}\;D+A$. The intermediate gateway then assigns the triple of (J,K,ℒ ) to underwater sensors.

The underwater sensors take the triplet (J,K,ℒ ) and data (𝓂) and generate an online signature. For this, it calculates ${𝒽}_{b}={ℋ}_{b}\left(ID,J,ℒ,\tau ,𝓂\;\right)$ and $\vartheta =𝒿+{𝒽}_{b}K\;mod\;n$. Finally, the underwater sensors compute the triple of ($ℒ,{𝒽}_{b},\vartheta$) as a full signature and send it to the surface user.

### 4.3. Signature Verification

The surface user can verify the signature triple ($ℒ,{𝒽}_{b},\vartheta$) on 𝓂 by computing ${𝒽}_{a}={ℋ}_{a}\left(ID,A\right)$, computing $J^{\prime }=\vartheta \;D-{𝒽}_{b}\left(ℒ+{𝒽}_{a}\;G\;\right)$, and computing ${𝒽}_{b}{}^{\prime }={ℋ}_{b}\left(ID,J,ℒ,\tau ,𝓂\;\right)$. The surface user then compares both ${𝒽}_{b}{}^{\prime }={𝒽}_{b}$; If it holds, the signature is considered legitimate; if not, it is considered to be forged.

## 5. Security Analysis

### 5.1. Theorem 1

**Definition** **3.** *“Under the security assumptions of the random oracle model (ROM), an adversary (* $T_{1}$ *) is unforgeable against the adaptive chosen message and identity attacks without knowledge of the partial private key and secret value.”*

**Proof.**  Assume (D, ℴD) as a random HCDLP stance that outputs 𝓸. An algorithm (Aℓ) will perform the subsequent simulations for interacting with $T_{1}$. □

**Setup:** In this phase, Aℓ performs the following steps.

1. The Aℓ sets the public key as  G= ℴ.D and advertises $W=\{\mathrm{HCC},{ℋ}_{a},{ℋ}_{b},G,n,D$} in the entire network.
2. For $1\leq 𝓅\leq Q_{{ℋ}_{a}}$, the Aℓ chooses ${\mathrm{ID}}_{p}$ at random as a challenging ID for this particular game, while $Q_{{ℋ}_{a}}$ represents the utmost number of the ${ℋ}_{a}$ querying oracle.
3. The Aℓ picks ${𝒻}_{p}\;\in \left\{𝟙,𝟚,𝟛,\ldots ,\left(𝕟-𝟙\right)\right\}$ at random and sets $A_{p}=-{𝒻}_{p}\left(ℴ.D\right)$, defines $C_{p}={ℋ}_{a}\left(ID,A\right)$, and adds the triple of (${\mathrm{ID}}_{p},A_{p},{𝒻}_{p}$) to the ${ℋ}_{a}{}^{list}$.
4. Finally, the Aℓ gives $T_{1}$ the global parameters set as $W=\{\mathrm{HCC},{ℋ}_{a},{ℋ}_{b},G,n,D$}.
5. After that, the Aℓ starts answering the queries from $T_{1}$ as

${𝓗}_{a}\;Queries$**:** The $T_{1}$ inputs (${\mathrm{ID}}_{i},A_{i}$), and with that, the Aℓ calls the ${ℋ}_{a}{}^{list}$. If the ${ℋ}_{a}{}^{list}$ has the (${\mathrm{ID}}_{i},A_{i},{𝒻}_{i}$), Aℓ provides it to the $T_{1}$. If not, the Aℓ picks ${𝒻}_{i}\;\in \left\{𝟙,𝟚,𝟛,\ldots ,\left(𝕟-𝟙\right)\right\}\;$ at random and adds (${\mathrm{ID}}_{i},A_{i},{𝒻}_{i}$) to the ${ℋ}_{a}{}^{list}$ and response $C_{i}$ to the $T_{1}$.

${𝓗}_{b}\;Queries:$ The $T_{1}$ inputs $\left(ID_{i},J_{i},{ℒ}_{i},{𝓂}_{i}\;\right),$ and with that, the Aℓ calls the ${ℋ}_{b}{}^{list}$. If the ${ℋ}_{b}{}^{list}$ already has the requested query, it simply returns back to the $T_{1}$. If not, the Aℓ picks ${𝒽}_{i}\;\in \left\{𝟙,𝟚,𝟛,\ldots ,\left(𝕟-𝟙\right)\right\}$ at random and adds $\left(ID_{i},J_{i},{ℒ}_{i},\tau ,{𝓂}_{i},{𝒽}_{i}\right)$ to the ${ℋ}_{b}{}^{list}$ and response ${𝒽}_{i}$ to the $T_{1}$.

**Partial Private Key Extraction Queries:** Upon requesting the private key associated with ${\mathrm{ID}}_{i}$, the Aℓ first verifies if ${\mathrm{ID}}_{i}={\mathrm{ID}}_{p}$ stays or not. The Aℓ also maintains the $Ext^{list}.$

1. If ${\mathrm{ID}}_{i}={\mathrm{ID}}_{p},\;$ the Aℓ terminates the simulation and outputs an error.
2. If ${\mathrm{ID}}_{i}\neq {\mathrm{ID}}_{p}$, the Aℓ choose $V_{ID}{}_{i}\in \left\{𝟙,𝟚,𝟛,\ldots ,\left(𝕟-𝟙\right)\right\}$ at random as of the secret value allied with ${\mathrm{ID}}_{i}$. The Aℓ picks $U_{ID}{}_{i}\;\in \left\{𝟙,𝟚,𝟛,\ldots ,\left(𝕟-𝟙\right)\right\}$ and computes ${ℒ}_{i}=U_{ID}{}_{i}.D+V_{ID}{}_{i}.D-{𝒻}_{i}\;ℴ.D$. If the ${ℋ}_{a}$(${\mathrm{ID}}_{i},A_{i},{𝒻}_{i}$) already exists, then the Aℓ terminates the simulation and outputs an error. The process is termed the Event by ${\mathrm{EVE}}_{1}$. The Aℓ then adds (${\mathrm{ID}}_{i},A_{i},{𝒻}_{i}$) and (${\mathrm{ID}}_{i},U_{ID}{}_{i},V_{ID}{}_{i}$) to the $Ext^{list}.$ To end with, the Aℓ outputs ${ℒ}_{i}$ and $U_{ID}{}_{i}$.

The probability of ${\mathrm{EVE}}_{1}$ is the utmost $\frac{\left(Q_{{ℋ}_{a}}+Q_{E}\right)}{2^{⋋+1}}$, where $Q_{E}$ represent the querying of the key extraction oracle. 

Secret Value Extraction Queries:

1. If ${\mathrm{ID}}_{i}={\mathrm{ID}}_{p},\;$ the Aℓ terminates the simulation and outputs an error.
2. If ${\mathrm{ID}}_{i}\neq {\mathrm{ID}}_{p}$, the Aℓ searches (${\mathrm{ID}}_{i},U_{ID}{}_{i},V_{ID}{}_{i}$) from the $Ext^{list}$ and responds to the allied secret value ($V_{ID}$).

**Signature Generation Queries:** Suppose a query for a signature with an identity (ID) and message (𝓂).

1. If ${\mathrm{ID}}_{i}={\mathrm{ID}}_{p},\;$ the Aℓ picks $\vartheta_{p}$,${𝒽}_{p}\in \left\{𝟙,𝟚,𝟛,\ldots ,\left(𝕟-𝟙\right)\right\}$ at random and sets ${ℒ}_{p}=\;ℴ.D-C_{p}\left(ℴ.D\right)$ and computes $J_{p}=\vartheta_{p}.D-{𝒽}_{p}\left({ℒ}_{p}+C_{p}G\right)$, where ${ℋ}_{b}\left(ID_{p},J_{p},{ℒ}_{p},\tau ,{𝓂}_{i}\right)$. If ${ℋ}_{b}\left(ID_{p},J_{p},{ℒ}_{p},{𝓂}_{i}\right)$ already exists, Aℓ terminates the simulation and outputs an error. The process is the Event ${\mathrm{EVE}}_{2}$.
2. Finally, the Aℓ outputs the triple (${ℒ}_{p},{𝒽}_{p}$,$\;\vartheta_{p}$) as the signature. The probability of ${\mathrm{EVE}}_{2}$ is utmost $\frac{\left(Q_{{ℋ}_{a}}+Q_{Sig}\right)}{2^{⋋}}$, where $Q_{Sig}$ represents the querying of the signature generation oracle.
3. If ${\mathrm{ID}}_{i}\neq {\mathrm{ID}}_{p}$, the signature is normal, as the Aℓ has the partial private key and secret value. Thus, the Aℓ can ordinarily perform the online signature generation.

**Forgery:** Let the $T_{1}$ generate a forgeable digital signature (${ℒ}^{*},{𝒽}^{*},\vartheta^{*}$) on the message (${𝓂}^{*}$) for a given identity (${\mathrm{ID}}^{*}$), though ${\mathrm{ID}}^{*}$ is not submitted to the secret value extraction oracle and partial private key extraction oracle, and (${𝓂}^{*}$,${\mathrm{ID}}^{*}$) is not a query to the signature generation oracle.

1. If ${\mathrm{ID}}^{*}\neq {\mathrm{ID}}_{p}{}^{*}$ and ${ℒ}^{*}\neq {ℒ}_{p}{}^{*}$, then the Aℓ terminates the simulation and outputs an error. The process is termed the Event ${\mathrm{EVE}}_{3}$. The probability of ${\mathrm{EVE}}_{2}$ is utmost $\frac{1}{Q_{{ℋ}_{a}}}$, where $Q_{{ℋ}_{a}}$ represent the utmost number of ${ℋ}_{a}$ querying the oracle.
2. If not, then according to the forking lemma [19], another algorithm (M) exists that is able to produce two valid digital signatures $\left(ID_{p},J_{p},{ℒ}_{p},{𝓂}^{*},{𝒽}_{1},\;\vartheta_{1}\right)$ and $\left(ID_{p},J_{p},{ℒ}_{p},\tau ,{𝓂}^{*},{𝒽}_{2},\;\vartheta_{2}\right)$ in a probabilistic polynomial time, where ${𝒽}_{1}\neq {𝒽}_{2}$ while $C_{p}$ remains the same due to $\left(ID_{p},A_{p}\right)={𝒻}_{p}$. Thus, the subsequent equations hold as
  $$J=\vartheta_{1}.D-{𝒽}_{1}\left({ℒ}_{p}+{𝒻}_{p}G\right)$$
  $$J=\vartheta_{2}.D-{𝒽}_{2}\left({ℒ}_{p}+{𝒻}_{p}G\right)$$

After the calculations, we obtain $\left(\vartheta_{1}-\vartheta_{2}\right)D=\left({𝒽}_{1}-{𝒽}_{2}\right)ℴ.D$, then get $ℴ=\left(\vartheta_{1}-\vartheta_{2}\right)/\left({𝒽}_{1}-{𝒽}_{2}\right)$ and output ℴ as a solution for the HCDLP instance, respectively.

### 5.2. Theorem 2

**Definition** **4.** *There is an adversary (* $T_{2}$ *) who is existentially unforgeable against the adaptive chosen message and identity attacks and has the knowledge of the partial private key/master secret key but does not have the participant’s secret value in the ROM under the security HCDLP assumptions.*

**Proof.**  Assume (D, ℴD) as a random HCDLP stance that outputs 𝓸. An algorithm (Aℓ) will perform the subsequent simulations for interacting with $T_{2}$. □

**Setup:** In this phase, Aℓ performs the following steps.

1. The Aℓ sets the public key as  G= ℴ.D and advertises $W=\{\mathrm{HCC},{ℋ}_{a},{ℋ}_{b},G,n,D$} in the entire network.
2. For $1\leq 𝓅\leq Q_{{ℋ}_{a}}$, the Aℓ chooses ${\mathrm{ID}}_{p}$ at random as a challenging ID for this particular game, while $Q_{{ℋ}_{a}}$ represents the utmost number of ${ℋ}_{a}$ querying oracles.
3. Finally, the Aℓ gives $T_{2}$ the global parameters set $W=\{\mathrm{HCC},{ℋ}_{a},{ℋ}_{b},G,n,D$} and master secret key (N).

After that, the Aℓ starts answering the queries from $T_{2}$ as:

${𝓗}_{a}\;Queries$**:** The $T_{2}$ inputs (${\mathrm{ID}}_{i},A_{i}$), and with that, the Aℓ calls the ${ℋ}_{a}{}^{list}$. If the ${ℋ}_{a}{}^{list}$ has the (${\mathrm{ID}}_{i},A_{i},{𝒻}_{i}$), Aℓ provides it to the $T_{2}$. If not, the Aℓ picks ${𝒻}_{i}\;\in \left\{𝟙,𝟚,𝟛,\ldots ,\left(𝕟-𝟙\right)\right\}$ at random and adds (${\mathrm{ID}}_{i},A_{i},{𝒻}_{i}$) to the ${ℋ}_{a}{}^{list}$ and response ${𝒻}_{i}$ to the $T_{2}$.

${𝓗}_{b}\;Queries:$ The $T_{2}$ inputs $\left(ID_{i},J_{i},{ℒ}_{i},\tau ,{𝓂}_{i}\right)$, and with that, the Aℓ calls the ${ℋ}_{b}{}^{list}$. If the ${ℋ}_{b}{}^{list}$ already has the requested query, it simply returns back to the $T_{2}$. If not, the Aℓ picks ${𝒽}_{i}\;\in \left\{𝟙,𝟚,𝟛,\ldots ,\left(𝕟-𝟙\right)\right\}$ at random and adds $\left(ID_{i},J_{i},{ℒ}_{i},\tau ,{𝓂}_{i},{𝒽}_{i}\right)$ to the ${ℋ}_{b}{}^{list}$ and response ${𝒽}_{i}$ to the $T_{2}$.

**Partial Private Key Extraction Queries:** Upon requesting the private key associated with ${\mathrm{ID}}_{i}$, the Aℓ first verifies if ${\mathrm{ID}}_{i}={\mathrm{ID}}_{p}$ stays or not. The Aℓ also maintains the $Ext^{list}.$

1. If ${\mathrm{ID}}_{i}={\mathrm{ID}}_{p},\;$ the Aℓ sets $A_{i}=ℴ.D$ and obtains (${\mathrm{ID}}_{i},A_{i},{𝒻}_{i}$) from ${ℋ}_{a}{}^{list}$. The Aℓ then picks ${𝒾}_{i}$ at random and computes $U_{ID}{}_{i}={𝒾}_{i}+N{𝒽}_{i}$ and adds (${\mathrm{ID}}_{i},U_{ID}{}_{i},⊥$) to the list (${\mathrm{ID}}_{i},U_{ID}{}_{i},{𝒾}_{i}$), where ⊥ represents the unknown secret value for the identity ${\mathrm{ID}}_{i}$. To end with, the Aℓ returns $U_{ID}{}_{i}$.
2. If ${\mathrm{ID}}_{i}\neq {\mathrm{ID}}_{p}$, the Aℓ finds (${\mathrm{ID}}_{i},A_{i},{𝒻}_{i}$) from the ${ℋ}_{a}{}^{list}$. The Aℓ then chooses ${𝒾}_{i1},{𝒾}_{i2}\in \left\{𝟙,𝟚,𝟛,\ldots ,\left(𝕟-𝟙\right)\right\}$ at random and computes $U_{ID}{}_{i}={𝒾}_{i2}+N{𝒻}_{i}$ and adds (${\mathrm{ID}}_{i},U_{ID}{}_{i},\;{𝒾}_{i1}$) to the list. To end with, the Aℓ returns $U_{ID}{}_{i}$

**Signature Generation Queries:** Suppose a $T_{2}$ query for a signature with an identity (ID) and message (𝓂).

1. If ${\mathrm{ID}}_{i}={\mathrm{ID}}_{p},\;$ the Aℓ picks $\vartheta_{i}$,${𝒽}_{i}\;\in \left\{𝟙,𝟚,𝟛,\ldots ,\left(𝕟-𝟙\right)\right\}$ at random and sets $A_{i}=ℴ.D$ and finds (${\mathrm{ID}}_{i},A_{i},{𝒻}_{i}$) from ${ℋ}_{a}{}^{list},$ and additionally, the Aℓ also sets ${ℒ}_{i}=A_{i}=ℴ.D$ and computes $J_{i}=\vartheta_{i}.D-{𝒽}_{i}\left({ℒ}_{i}+{𝒻}_{i}G\right)$, where ${𝒽}_{i}={ℋ}_{b}\left(ID_{i},J_{i},{ℒ}_{i},\tau ,{𝓂}_{i}\right)$. If ${ℋ}_{b}\left(ID_{i},J_{i},{ℒ}_{i},{𝓂}_{i}\right)$ already exists, Aℓ terminates the simulation and outputs an error. The process is termed the Event ${\mathrm{EVE}}_{2}$.
  - Computes $J_{p}=\vartheta_{p}.D-{𝒽}_{p}\left({ℒ}_{p}+{𝒻}_{p}G\right)$, where ${ℋ}_{a}\left(ID_{p},J_{p},{ℒ}_{p},\tau ,{𝓂}_{i}\right)$. If ${ℋ}_{a}\left(ID_{p},J_{p},{ℒ}_{p},\tau ,{𝓂}_{i}\right)$ already exists, Aℓ terminates the simulation and outputs an error. The process is termed the Event ${\mathrm{EVE}}_{2}$. Finally, the Aℓ outputs the triple $\left({ℒ}_{i},{𝒽}_{i},\;\vartheta_{i}\right)$ as the signature. The probability of ${\mathrm{EVE}}_{2}$ is the utmost $\frac{\left(Q_{{ℋ}_{b}}+Q_{Sig}\right)}{2^{⋋}}$, where $Q_{Sig}$ represents the querying of the signature generation oracle.
2. If ${\mathrm{ID}}_{i}\neq {\mathrm{ID}}_{p}$, the signature is normal, as the Aℓ has the partial private key and secret value. Thus, the Aℓ can ordinarily perform the online signature generation.

**Forgery:** Let the $T_{2}$ generate a forgeable digital signature (${ℒ}^{*},{𝒽}^{*},\vartheta^{*}$) on the message (${𝓂}^{*}$) for a given identity (${\mathrm{ID}}^{*}$), though ${\mathrm{ID}}^{*}$ is not submitted to the secret value extraction oracle, and (${𝓂}^{*}$,${\mathrm{ID}}^{*}$) is not query to the signature generation oracle.

1. If ${\mathrm{ID}}^{*}\neq {\mathrm{ID}}_{p}{}^{*}$ and ${ℒ}^{*}\neq {ℒ}_{p}{}^{*}$, then the Aℓ terminates the simulation and outputs an error. The process is termed as the Event ${\mathrm{EVE}}_{3}$. The probability of ${\mathrm{EVE}}_{2}$ is not less than $\frac{1}{Q_{{ℋ}_{a}}}$, where $Q_{{ℋ}_{a}}$ represent the utmost number of ${ℋ}_{a}$ querying oracles.
2. If not, then according to the forking lemma [19], another algorithm (M) exists that is able to produce two valid digital signatures $\left(ID_{p},J,{ℒ}_{p},{𝓂}^{*},{𝒽}_{1},\;\vartheta_{1}\right)$ and $\left(ID_{p},J,{ℒ}_{p},{𝓂}^{*},{𝒽}_{2},\;\vartheta_{2}\right)$ in a probabilistic polynomial time, where ${𝒽}_{1}\neq {𝒽}_{2}$ and $A^{\prime }={ℒ}^{\prime }D$ ${𝒻}_{p}$ remain the same. Thus, the subsequent equations hold as:
  $$J=\vartheta_{1}.D-{𝒽}_{1}\left({ℒ}_{p}+{𝒻}_{p}G\right)$$
  $$J=\vartheta_{2}.D-{𝒽}_{2}\left({ℒ}_{p}+{𝒻}_{p}G\right)$$

After the calculations, we obtain $\left(\vartheta_{1}-\vartheta_{2}\right)D=\left({𝒽}_{1}-{𝒽}_{2}\right)\left(ℴ+N{𝒻}_{p}\right)D$, then get $ℴ=\frac{\left(\vartheta_{1}-\vartheta_{2}\right)}{({𝒽}_{1}-{𝒽}_{2)}}-N{𝒻}_{p}$ and output ℴ as a solution for the HCDLP instance, respectively.

### 5.3. Theorem 3

**Definition** **5.** *If the NM impersonates an authentic participant in order to forge the signature and has knowledge of the participant’s partial private key and secret value (an alternate secret value that is not real), we can demonstrate to the mediator that the NM is dishonest.*

**Proof.**  According to the above two theorems, the proposed scheme is unforgeable against both malicious type-1 and type-2 adversaries. The process is split into two steps, i.e., forging the private key and signing the message. □

**Forging the Private Key:** Let ID be the identity of the participant, and ($V_{ID},U_{ID}$) is the respective private key. The NM simulates the participant to generate a signature in two possible ways:

1. By knowing the participant’s secret value $V_{ID}$.
2. By replacing the participant’s secret value $V_{ID}$. As we know that the $V_{ID}$ is picked at random from the {𝟙,𝟚,𝟛,…,(𝕟−𝟙)}, it is infeasible of the NM to obtain the $V_{ID}$.

Thus, the NM has to pick a secret value $V_{ID}$ for the participants to produce another private key using the identity ID. The procedure is mentioned below.

1. The NM picks $V_{ID}$ for the replacement of the participant’s secret value.
2. The NM picks ${𝒾}^{\prime }\;\in \left\{𝟙,𝟚,𝟛,\ldots ,\left(𝕟-𝟙\right)\right\}\;$ at random and computes $A^{\prime }\;={𝒾}^{\prime }.D$ and $U_{ID}{}^{\prime }={𝒾}^{\prime }+N{𝒻}_{p}{}^{\prime }mod\;n$. Let $A^{\prime }$,$\;U_{ID}{}^{\prime }$ satisfy and produce a private key ($V_{ID}{}^{\prime },\;U_{ID}{}^{\prime }$).

**Signing message:** After forging the participant private key ($V_{ID}{}^{\prime },\;U_{ID}{}^{\prime }$), the NM executes the signature generation algorithm. The triple (${𝒾}^{\prime },{𝒽}^{\prime },\vartheta^{\prime }$) on the message 𝓂 is for a given identity (ID) of the participant. The participant can run the signature generation algorithm twice to make sure that (${𝒾}^{\prime },{𝒽}^{\prime },\vartheta^{\prime }$) is forged by the NM or an adversary conspired with the NM. Let the participant produce two signatures, ($ℒ,{𝒽}_{1},\vartheta_{1}$) and ($ℒ,{𝒽}_{2},\vartheta_{2}$), and submit the ($ℒ,{𝒽}_{1},\vartheta_{1}$) and ($ℒ,{𝒽}_{2},\vartheta_{2}$) to the intermediary trusted authority.

Note: Here, ${ℒ}^{\prime }\neq ℒ$,. If the NM aims to make ${ℒ}^{\prime }=ℒ,$ then the NM needs to satisfy $({𝒾}^{\prime }+V_{ID}{}^{\prime })D=\left(𝒾+V_{ID}\right)D$. Furthermore, the NM also needs to know the value $A^{\prime }=\left(𝒾+V_{ID}-V_{ID}{}^{\prime }\right)D={𝒾}^{\prime }D$, but the NM does not know about $V_{ID}$. Thus, according to the HCDLP, it is infeasible for the NM to obtain $𝒾,{𝒻}_{p}$ and $U_{ID}$. Hence, ${ℒ}^{\prime }\neq ℒ$.

Now, if the above three signatures are valid, then the ℒ in the triple ($ℒ,{𝒽}_{1},\vartheta_{1}$) and ($ℒ,{𝒽}_{2},\vartheta_{2}$) are the same. We obtain ${ℒ}^{\prime }\neq ℒ$ in (${ℒ}^{\prime },{𝒽}^{\prime },\vartheta^{\prime }$). Hence, (${ℒ}^{\prime },{𝒽}^{\prime },\vartheta^{\prime }$) definitely is forged by the NM or an adversary conspired with the NM.

## 6. Cost Efficiency

Here, we compared the proposed certificateless online/offline signature scheme with previously suggested online/offline signature schemes based on the communication bandwidth and computation time.

### 6.1. Computation Time

The proposed scheme is compared with some of the most recent online/offline signature schemes, i.e., Addobea et al. [19], Dan et al. [20], Khan et al. [22], and Hong et al. [24], in order to evaluate how well it performs in terms of the amount of computation that is required. A MIRACLE “C” Library [31] used to evaluate the effectiveness of the proposed strategies in light of the costly mathematical operations. For testing the simulation results, a device with the features used is stated in Table 3 [27]. The key operation of our comparative analysis is explained in Table 4, Table 5 and Table 6, respectively. For our comparative analysis, we consider the costly mathematical operations pairing operations (𝒫𝒪), bilinear pairing scalar multiplication (𝒫ℬ𝒮ℳ), ECC-based scalar multiplication (ℰℬ𝒮ℳ), and hyperelliptic curve devisor multiplication (ℋ𝒞𝒟ℳ). Previous observations show that the running processing time of a single point multiplication varies significantly: ℰℬ𝒮ℳ takes 0.83 ms, 𝒫𝒪 consumes 20.01 ms, and 𝒫ℬ𝒮ℳ consumes 6.38 ms [32]. Owing to the 80-bit key size, ℋ𝒞𝒟ℳ is estimated to be half of ECC, so it will consume 0.415 ms [22].

**Figure 5:**
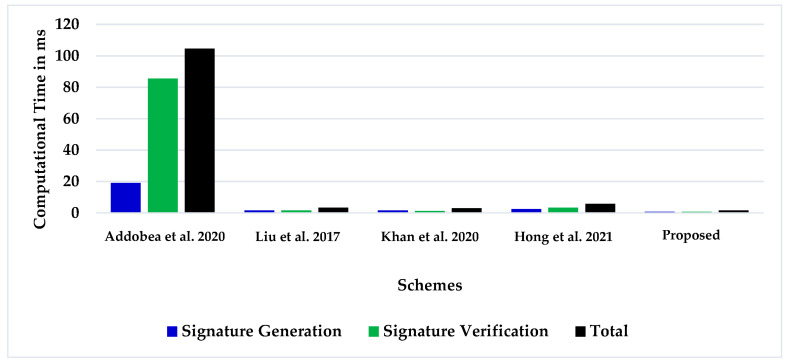
Computation time evaluation [19,20,22,24].

The sender of the message executes the certificateless online/offline signature generation algorithm of the proposed scheme, which involves  two ℋ𝒞𝒟ℳ to produce the certificateless online/offline signature. Additionally, the certificateless online/offline signature verifier requires two ℋ𝒞𝒟ℳ to authenticate the online/offline signature. Table 4 shows the computation time required by the suggested online/offline cryptographic schemes in terms of costly operations. Moreover, Table 5 demonstrates the efficiency evaluation comparison between the proposed scheme and the previous design schemes in milliseconds. According to Table 6, the essential time-designed scheme is almost 98.41% of Addobea et al. [20], 50%  of Liu et al. [19], 42.85% of Khan et al. [22], and 71.42% of Hong et al. [24]. Additionally, Figure 5 demonstrates the computational time evaluation analysis of certificateless online/offline signature generation and verification. The vertical axis indicates the computation time in milliseconds for a clear representation of the computation timeframe. It is obvious that the new strategy is more effective than the previous.

**Percentage Improvement in terms of the Computation Time.**

The computation time improvement is shown in Table 7 below.

### 6.2. Communication Overhead

**Figure 6:**
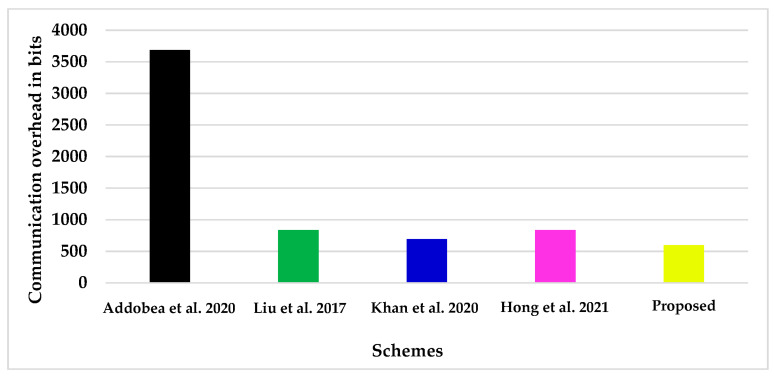
Communication overhead evaluation [19,20,22,24].

Specifically, we compare the proposed scheme with a few recent online/offline signature schemes, including those presented by Addobea et al. [20], Liu et al. [19], Khan et al. [22], and Hong et al. [24], in order to illustrate how the designed approach is more efficient in terms of the communication overhead. In order to do so, we assume that the length of elements in |G1| = |G2| = |G| = 1024 bits for bilinear pairing, |q| = 160 bits for the elliptic curve cryptosystem, |n| = 80 bits for the hyperelliptic curve cryptosystem, |m| = 100 bits, and |H| = 256 for the hash function [33]. Furthermore, Table 8 and Table 9 depict the percentage improvement in the communication overhead that may be achieved by using the designed technique. Additionally, Figure 6 shows the results of an examination of the communication overhead of the certificateless online/offline signature systems. The vertical axis depicts the communication overhead in bits, which allows for a clear visual representation of the communication overhead. It demonstrates unequivocally that the designed strategy is more efficient than the previously designed approaches.

**Percentage Improvement in terms of the communication overhead**

The communication overhead improvement is shown in Table 9 below.

### 6.3. Performance Evaluation Using EDAS

EDAS is a standard approach that is utilized for testing and evaluating a variety of alternative options. Gorhabaee et al. [34] were the first people to apply the approach. The Positive Distance from Average and Negative Distance from Average solutions are the two functions that are used in EDAS to measure how far a solution is from the average [35]. EDAS is a multi-criteria decision-making (MCDM) approach that calculates the distance of all other solutions from the average solution and uses that specific information to select the best among the alternatives [36].

The EDAS is generally selected for a comparative analysis in a situation to solve the conflicting criteria [30]. Table 10 shows a comparative analysis of the selected performance metrics. In addition, the EDAS technique is used to select the most effective values for the four different methods, depending on the selected parameters.

Furthermore, the assessment scores (μ) were used to calculate the ranking based on the chosen parameters among the existing schemes. Table 10 evaluates the performance matrices of the previously proposed schemes, including ours.

***Step One (Average Solution):***

In this step, the average of the selected matrices is calculated.

(1) $$\left(\phi \right)={\left[\vartheta_{b}\right]}_{1\times \beta }$$

while

(2) $$=\frac{\sum_{i=1}^{y}X_{ab}}{y}$$

In the stage before this one, one of the criteria for determining which solution to recommend is the performance of the matrices that were chosen. Precisely, in this step, the average of the selected matrices is calculated. As can be seen in Table 11, each calculated value on a chosen matrix can be derived as a solution to Equations (1) and (2), respectively.

***Step Two: Positive Distance from Average (*** $𝓟{𝓓}_{𝓪𝓿𝓰}$ ***)***

In this step, the $P_{dav}$ is calculated using the following equations:

(3) $${𝒫𝒟}_{𝒶𝓋ℊ}={\left[{\left({𝒫𝒟}_{𝒶𝓋ℊ}\right)}_{ab}\right]}_{\beta \times \beta }$$

If the state $b^{th}$ is favorable, then

(4) $${\left({𝒫𝒟}_{𝒶𝓋ℊ}\right)}_{ab}=\frac{ℳAX\left(0,\left(Ave_{b}-X_{ab}\right)\right)}{Ave_{b}}$$

For the less favorable, it becomes

(5) $${\left({𝒫𝒟}_{𝒶𝓋ℊ}\right)}_{ab}=\frac{ℳAX\left(0,\left(X_{ab}-Ave_{b}\right)\right)}{Ave_{b}}$$

where ${𝒫𝒟}_{𝒶𝓋ℊ}$ represents the Positive Distance from Average from the given average value on the $a^{th}$ rating performance matrices.

***Step Three: Negative Distance from Average (*** $𝓝{𝓓}_{𝓪𝓿𝓰}$ ***)***

The ${𝒩𝒟}_{𝒶𝓋ℊ}$ is calculated in this step using the following equations:

(6) $$\left({𝒩𝒟}_{𝒶𝓋ℊ}\right)={\left[{\left({𝒩𝒟}_{𝒶𝓋ℊ}\right)}_{ab}\right]}_{\beta \times \beta }$$

If the $b^{th}$ criterion is more favorable than

(7) $${\left({𝒩𝒟}_{𝒶𝓋ℊ}\right)}_{ab}=\frac{ℳAX\left(0,\left(Ave_{b}-X_{ab}\right)\right)}{Ave_{b}}$$

and less desirable, then the given above equations become

(8) $${\left({𝒩𝒟}_{𝒶𝓋ℊ}\right)}_{ab}=\frac{ℳAX\left(0,\left(X_{ab}-Ave_{b}\right)\right)}{Ave_{b}}$$

where ${\left({𝒩𝒟}_{𝒶𝓋ℊ}\right)}_{ab}$ represents the Negative Distance from Average solution.

***Step Four: Weighted Sum of the Positive Distance (*** ${𝓦𝓢𝓟𝓓}_{𝓪𝓿𝓰}$ ***)***

The ${𝒲𝒮𝒫𝒟}_{𝒶𝓋ℊ}$ for the given schemes are considered at this stage, as shown in Table 12.

(9) $${𝒲𝒮𝒫𝒟}_{𝒶𝓋ℊ}=\sum_{b=1}^{y}\lambda_{b}{\left(𝒫𝒟\right)}_{ab}$$

***Step Five: The Weighted Sum of the Negative Distance (*** ${𝓦𝓢𝓝𝓓}_{𝓪𝓿𝓰}$ ***)***

For the ${𝒲𝒮𝒩𝒟}_{𝒶𝓋ℊ}$ for the selected scheme obtained in this phase employing the following formula, the results are shown in Table 13.

(10) $${𝒲𝒮𝒩𝒟}_{𝒶𝓋ℊ}=\sum_{b=1}^{y}\lambda_{b}{\left(𝒩𝒟\right)}_{ab}$$

***Step Six (Ranking)***

The scores that were generated based on the ${𝒲𝒮𝒫𝒟}_{𝒶𝓋ℊ}\;\mathrm{and}\;{𝒲𝒮𝒩𝒟}_{𝒶𝓋ℊ}$, are presented accordingly in the following Equations (11) and (12).

(11) $$N\left({𝒲𝒮𝒫𝒟}_{𝒶𝓋ℊ}\right)=\frac{{𝒲𝒮𝒫𝒟}_{𝒶𝓋ℊ}}{ℳAX_{a}\left({𝒲𝒮𝒫𝒟}_{𝒶𝓋ℊ}\right)}$$

(12) $$\begin{array}{c} N({𝒲𝒮𝒩𝒟}_{𝒶𝓋ℊ})=1-\frac{{𝒲𝒮𝒩𝒟}_{𝒶𝓋ℊ}}{ℳAX_{a}\left({𝒲𝒮𝒩𝒟}_{𝒶𝓋ℊ}\right)} \end{array}.\;$$

The score values based on $N({𝒲𝒮𝒩𝒟}_{𝒶𝓋ℊ})\;\mathrm{and}$ $N({𝒲𝒮𝒩𝒟}_{𝒶𝓋ℊ})$ are based on the evaluation scores (μ) for the rated schemes, as stated in Equation (13).

(13) $$\mu =\frac{1}{2}\left(N{𝒲𝒮𝒫𝒟}_{𝒶𝓋ℊ}-N{𝒲𝒮𝒩𝒟}_{𝒶𝓋ℊ}\right),\;\mathrm{where}\;0\;\leq \mu \geq \;1$$

We obtained the final result by utilizing both the ${𝒲𝒮𝒫𝒟}_{𝒶𝓋ℊ}\;\mathrm{and}$ ${𝒲𝒮𝒩𝒟}_{𝒶𝓋ℊ}$ average.

Following the steps outlined above establishes the extent of μ and provides the final ranking based on the parameters selected for the adopted schemes. According to the evaluation results, the best online/offline signature scheme obtains the highest scores. As may can be seen in Table 14, the proposed scheme has received very good evaluation scores (μ).

According to the conclusive findings of the EDAS technique, the overall performance of our scheme is superior than that of the earlier online/offline signature schemes. On the basis of a comparison study using fuzzy logic-based EDAS, the new scheme is superior to that of Khan et al. [22] and Liu et al. [19], which come in second and third, respectively. The Hong et al. [24] approach, on the other hand, comes in fourth place in the chosen matrix.

## 7. Summary of the Findings

To the best of our knowledge, we designed the first ever online/offline signature scheme for UWSNs. The proposed scheme makes the least possible use of computational and communicational resources by employing lightweight HEC. In addition to that, the proposed scheme uses the idea of online/offline signatures in order to lessen the load on the sensors nodes. A fuzzy-based EDAS technique was applied in the proposed system in order to illustrate both the practicability and effectiveness of the given approach. According to the results of the findings, the proposed scheme is superior in terms of the chosen parameters. Finally, an application shown where the proposed scheme is deployed.

## 8. Conclusions

The paper presents a lightweight certificateless online/offline signature scheme for underwater wireless sensor networks (UWSNs). The signature is completed in two stages, according to the proposed scheme, the first of which takes place online and the second of which takes place offline. In the absence of a message, the offline phase is responsible for carrying out computationally complex operations, whereas the online phase is responsible for carrying out computations that are more straightforward and less intensive. In addition to this, the proposed scheme utilized a lightweight hyperelliptic curve cryptosystem that has an 80-bit key size in order to bring down the overall cost of the UWSNs even further. Additionally, the newly proposed scheme is compared with the previously suggested online and offline signature schemes with regards to the amount of computation time and communication overhead. In comparison to the previous schemes, the proposed schemes minimize the amount of time needed for computation from 50% to 98.41% and reduces the amount of communication overhead from 13.87% to 83.82%. In addition, the proposed scheme is proven secure in the random oracle model under the hyperelliptic curve discrete logarithm problem. The feasibility of a proposed scheme is demonstrated by a security analysis and comparisons with the relevant current schemes. A decision-making strategy known EDAS was also used to demonstrate the design effectiveness in multiple criteria. Finally, we presented a scenario in which the proposed approach can be practically applied on underwater wireless sensor networks.

## Figures and Tables

**Table 2:** Notation table.

S/N	Definition	Notations
1	Security Parameter	ζ
2	Public Parameter Set	W
3	NM Master Key	G
4	Identity of Users	ID
5	Partial Private Key	$U_{i}$
6	Secret Value	$V_{i}$
7	Full Private Key	$(V_{i},U_{i}$)
8	Signature	$(ℒ,{𝒽}_{b},\vartheta$)
9	assessment scores	μ
10	Average Value	ϑ
11	Positive Distance from Average	${𝒫𝒟}_{𝒶𝓋ℊ}$
12	Negative Distance from Average	${𝒩𝒟}_{𝒶𝓋ℊ}$
13	Weighted Sum of the Positive Distance	${𝒲𝒮𝒫𝒟}_{𝒶𝓋ℊ}$
14	Negative Distance	𝒩𝒟
15	Weighted Sum of the Negative Distance	${𝒲𝒮𝒩𝒟}_{𝒶𝓋ℊ}$
16	Positive Distance	𝒫𝒟

**Table 3:** Hardware and software specifications.

System	Specification
Library	Multi-Precision Integer and Rational Arithmetic C Library
Hardware Processor	PIV 3 GHZ
RAM	512 MB
OS	Windows XP

**Table 4:** Computation of the costs of both online and offline signature generation.

Operations/Ref. No	Addobea et al. [20]	Liu et al. [19]	Khan et al. [22]	Hong et al. [24]	Proposed
Pairing Operations (**𝓟𝓞**)					
Bilinear Pairing Scalar Multiplication (**𝓟𝓑𝓢𝓜**)	3 𝓟𝓑𝓢𝓜				
ECC Based Scalar Multiplication (**𝓔𝓑𝓢𝓜**)		**𝟐 𝓔𝓑𝓢𝓜**		3 𝓟𝓑𝓢𝓜	
Hyperelliptic Curve Devisor Multiplication (**𝓗𝓒𝓓𝓜**)			**𝟒 𝓗𝓒𝓓𝓜**		2 𝓗𝓒𝓓𝓜
Total cost of Signature Generation	19.14 ms	1.66 ms	1.66 ms	2.49 ms	0.83 ms

**Table 5:** Computation of the costs of both online and offline signature verification.

**Operation/Ref. No**	Addobea et al. [20]	Liu et al. [19]	Khan et al. [22]	Hong et al. [24]	Proposed
Pairing Operations (**𝓟𝓞**)	3 𝒫𝒪				
Bilinear Pairing Scalar Multiplication (**𝓟𝓑𝓢𝓜**)	4 𝒫ℬ𝒮ℳ				
ECC Based Scalar Multiplication (**𝓔𝓑𝓢𝓜**)		2 ℰℬ𝒮ℳ		4 ℰℬ𝒮ℳ	
Hyperelliptic Curve Devisor Multiplication (**𝓗𝓒𝓓𝓜**)			3 ℋ𝒞𝒟ℳ		2 ℋ𝒞𝒟ℳ
Total Signature Verification Time	85.55 ms	1.66 ms	1.245 ms	3.32 ms	0.83 ms

**Table 6:** Total computation costs of both the online and offline phases.

Operation/Ref. No	Addobea et al. [20]	Liu et al. [19]	Khan et al. [22]	Hong et al. [24]	Proposed
Pairing Operations (𝒫𝒪)	3 𝒫𝒪				
Bilinear Pairing Scalar Multiplication (𝒫ℬ𝒮ℳ)	7 𝒫ℬ𝒮ℳ				
ECC Based Scalar Multiplication (ℰℬ𝒮ℳ)		4 ℰℬ𝒮ℳ		7 ℰℬ𝒮ℳ	
Hyperelliptic Curve Devisor Multiplication (ℋ𝒞𝒟ℳ)			7 ℋ𝒞𝒟ℳ		4 ℋ𝒞𝒟ℳ
Total Computation Time	3 𝒫𝒪+7 𝒫ℬ𝒮ℳ = 104.69 ms	4 ℰℬ𝒮ℳ=3.32 ms	7 ℋ𝒞𝒟ℳ = 2.905 ms	7 ℰℬ𝒮ℳ= 5.81 ms	4 ℋ𝒞𝒟ℳ = 1.66 ms

**Table 7:** Computation overhead improvement.

Ref. No.	Computation Cost of Previous Scheme in MS	Computation Cost of Proposed	Percentage Improvement
Addobea et al. [20]	104.69	1.66	98.41
Liu et al. [19]	3.32	1.66	50
Khan et al. [22]	2.905	1.66	42.85
Hong et al. [24]	5.81	1.66	71.42

**Table 8:** Efficiency analysis of the communication overhead.

Operation/Ref. No	Addobea et al. [20]	Liu et al. [19]	Khan et al. [22]	Hong et al. [24]	Proposed
Ciphertext Size	3|G|+|m|+2|ℋ|	3|𝓃|+|m|+1|ℋ|	2|q|+|m|+2|ℋ|	3|𝓃|+|m|+1|ℋ|	3|q|+|m|+1|ℋ|
Total communication overhead in bits	3684 bits	836 bits	692 bits	836 bits	596 bits

**Table 9:** Communication overhead improvement.

Ref. No.	CO of Previous Scheme in MS	CO of Proposed	Percentage Improvement
Addobea et al. [20]	3684	596	83.82
Liu et al. [19]	836	596	28.70
Khan et al. [22]	692	596	13.87
Hong et al. [24]	836	596	28.70

**Table 10:** Performance metrics of the suggested schemes.

Weightage	0.25	0.25	0.25	0.25
Ref. NO.	Computation Overhead (ms)	Communication Overhead (bits)	Security (Yes/NO)	Computational and Communicational Efficiency (Yes/NO)
Addobea et al. [20]	104.69	3684	1	0
Liu et al. [19]	3.32	836	1	0.5
Khan et al. [22]	2.905	692	0	1
Hong et al. [24]	5.81	836	1	0.5
Proposed	1.66	596	1	1

**Table 11:** Average of the selected matrices.

Ref. NO.	Computation Overhead (ms)	Communication Overhead (bits)	Security (Yes/NO)	Computational and Communicational Efficiency (0,0.5,1)
Addobea et al. [20]	104.69	3684	1	0
Liu et al. [19]	3.32	836	1	0.5
Khan et al. [22]	2.905	692	0	1
Hong et al. [24]	5.81	836	1	0.5
Proposed	1.66	596	1	1
Average	23.677	1328.8	0.8	0.6

**Table 12:** Weighted sum of the positive distance.

Ref. NO.	Computation Overhead (ms)	Communication Overhead (bits)	Security (Yes/NO)	Computational and Communicational Efficiency (Yes/NO)	$\;{𝓦𝓢𝓟𝓓}_{𝓪𝓿𝓰}$
Addobea et al. [20]	0	0	0.0625	0	0.0625
Liu et al. [19]	0.214944883	0.092715232	0.0625	0	0.37016012
Khan et al. [22]	0.219326773	0.119807345	0	0.166666667	0.50580078
Hong et al. [24]	0.188653546	0.092715232	0.0625	0	0.34386878
Proposed	0.232472442	0.137868754	0.0625	0.166666667	0.59950786

**Table 13:** Weighted sum of the negative distance.

Ref. NO.	Computation Overhead (ms)	Communication Overhead (bits)	Security (Yes/NO)	Computational and Communicational Efficiency (Yes/NO)	$\;{𝓦𝓢𝓝𝓓}_{𝓪𝓿𝓰}$
Addobea et al. [20]	0.855397643	0.443106562	0	0.25	1.54850421
Liu et al. [19]	0	0	0	0.041666667	0.04166667
Khan et al. [22]	0	0	0.25	0	0.25
Hong et al. [24]	0	0	0	0.041666667	0.04166667
Proposed	0	0	0	0	0

**Table 14:** Ranking under the selected parameters.

Ref. NO.	$\;{𝓦𝓢𝓟𝓓}_{𝓪𝓿𝓰}$	$\;{𝓦𝓢𝓝𝓓}_{𝓪𝓿𝓰}$	$\;𝓝({𝓦𝓢𝓟𝓓}_{𝓪𝓿𝓰})$	$\;𝓝({𝓦𝓢𝓝𝓓}_{𝓪𝓿𝓰})$	μ	Ranking
Addobea et al. [20]	0.0625	1.548504206	0.104252177	0.932675561	0.51846387	5
Liu et al. [19]	0.370160115	0.041666667	0.617439968	0.601266845	0.60935341	3
Khan et al. [22]	0.505800784	0.25	0.84369333	0.455155933	0.64942463	2
Hong et al. [24]	0.343868777	0.041666667	0.573585101	0.629587638	0.60158637	4
Proposed	0.599507862	0	1	0.354215509	0.67710775	1

## Data Availability

Not applicable.
